# The JUMPFOOD study: additional effect of hydrolyzed collagen and vitamin C to exercise treatment for patellar tendinopathy (jumper’s knee) in athletes—study protocol for a double-blind randomized controlled trial

**DOI:** 10.1186/s13063-023-07783-2

**Published:** 2023-11-28

**Authors:** L. van Dam, R. Terink, M. Mensink, R. J. de Vos, J. Zwerver

**Affiliations:** 1 Department of Sports Medicine, SportsValley, Hospital Gelderse Vallei, Willy Brandtlaan 10, 6716 RP Ede, The Netherlands; 2 Division of Human Nutrition and Health, Wageningen University & Research (WUR), Wageningen, The Netherlands; 3 Erasmus MC University Medical Center, Rotterdam, The Netherlands; 4 Center for Human Movement Sciences, University of Groningen, University Medical Center Groningen, Groningen, The Netherlands

**Keywords:** Patellar tendinopathy, Jumper’s knee, Hydrolyzed collagen, Vitamin C, Supplements, Progressive tendon loading exercise, Nutrition

## Abstract

**Background:**

Patellar tendinopathy (PT) is a common problem in jumping athletes. Management can be challenging and treatment outcome is not always successful. In combination with tendon loading exercises, hydrolyzed collagen/vitamin C supplementation appears to have a promising effect on the recovery of tendinopathy. The aim of this study is to evaluate whether the use of oral supplementation of hydrolyzed collagen and vitamin C in combination with progressive tendon loading exercises (PTLE) is superior to PTLE and placebo on VISA-P score (which rates pain, function, sports participation) after 24 weeks for athletes with PT.

**Methods:**

The JUMPFOOD study is a double-blinded, two-armed randomized controlled trial, in which the effectiveness of oral supplementation of hydrolyzed collagen/vitamin C combined with PTLE compared to PTLE with placebo on pain and recovery of function in athletes with PT will be investigated. Seventy-six athletes aged 16–40 years, with symptoms of PT for at least 12 weeks, who play sports at least once a week will be included. All participants will receive education, advice with regard to load management and a PTLE program according to the Dutch guidelines for anterior knee pain. In addition, the intervention group will receive daily 10 g hydrolyzed collagen and 40 mg vitamin C supplementation for 24 weeks whereas the control group receives 10 g maltodextrin placebo supplementation. Measurements will take place at baseline and at 12 and 24 weeks’ follow-up. Primary outcome is the VISA-P score, which evaluates pain, function, and sports participation. For secondary outcome measures, data with regard to pain during functional tests, flexibility measurements, blood withdrawals, imaging characteristics of the tendon, and health questionnaires will be collected. During the follow-up period, participants will register sports participation, amount of training and tendon load, pain during sports, co-medication, and side-effects in a digital weekly diary.

**Discussion:**

The JUMPFOOD study is the first large RCT to study the effectiveness of hydrolyzed collagen/vitamin C supplementation in combination with the PTLE program in athletes with patellar tendinopathy. If supplementation of collagen/vitamin C appears to be effective, this treatment can be implemented in daily sports medicine practice to improve the treatment outcome of patients with PT.

**Trial registration:**

ClinicalTrials.gov NCT05407194. Registered on 7 June 2022.

**Supplementary Information:**

The online version contains supplementary material available at 10.1186/s13063-023-07783-2.

## Administrative information

Note: the numbers in curly brackets in this protocol refer to SPIRIT checklist item numbers. The order of the items has been modified to group similar items (see http://www.equator-network.org/reporting-guidelines/spirit-2013-statement-defining-standard-protocol-items-for-clinical-trials/).

**Table:** 

Title {1}	The JUMPFOOD study: Additional effect of hydrolyzed collagen and vitamin C to exercise treatment for Patellar Tendinopathy (Jumper’s knee) in athletes; Study protocol for a double blind randomized controlled trial
Trial registration {2a and 2b}.	Clinical trials registry (www.clinicaltrials.gov): NCT05407194 Trial registration number for medical ethical committee Oost-NL: NL79100.091.22
Protocol version {3}	Version 1, 20–04-2023
Funding {4}	This study was financially supported by the research fund of the Gelderse Vallei Hospital in Ede, the Netherlands, and by Rousselot inc. (Ghent, Belgium).
Author details {5a}	L van Dam^1,2^, R Terink^1^, M Mensink^2^, R de Vos^3^, J Zwerver^1,4^ ^1^SportsValley, Department of Sports Medicine, Hospital Gelderse Vallei, Willy Brandtlaan 10, 6716 RP, Ede, the Netherlands. ^2^Division of Human Nutrition and Health, Wageningen University & Research (WUR), Wageningen, the Netherlands. ^3^Erasmus MC University Medical Center, Rotterdam, The Netherlands. ^4^Center for Human Movement Sciences, University of Groningen, University Medical Center Groningen, The Netherlands
Name and contact information for the trial sponsor {5b}	Hospital Gelderse Vallei (ZGV) Willy Brandtlaan 10 6716 RP, Ede, The Netherlands www.wetenschapsbureau@zgv.nl
Role of sponsor {5c}	Sponsor: The hospital supported the JUMPFOOD study by providing a financial compensation for the execution of the study. Within the hospital, the study has been designed, prepared and will be executed. Funder: Rousselot supported the JUMPFOOD study by providing the intervention and placebo supplements and provided a financial compensation for the execution of the study. Rousselot has not been involved in the design of the study, nor will it be involved in collection, management, analysis and interpretation of the data and in publications. The investigators will have the ultimate authority over any of these activities.

## Introduction

### Background and rationale {6a}

Patellar tendinopathy (PT) is a tendon overuse injury that is clinically recognized by load-related pain localized at the inferior patellar pole [1]. Prevalence rates of up to 45% have been reported in elite athletes who participate in sports with repetitive jumping and landing kinetics, such as basketball and volleyball [2]. PT can result in a prolonged absence from sports participation, hampering individuals to achieve their desired performance levels [3]. The pathophysiology and multifactorial etiology of PT have not been elucidated so far; however, (over)load seems to be a driving factor [4]. Education, load management advice, and progressive tendon loading exercises (PTLE) are considered the preferential treatment to improve tendon symptoms, function, and structure [5–7].

Next to the effects of loading on tendon health, nutritional interventions can positively affect collagen synthesis in musculoskeletal tissues [8]. Supplementing 15 g of hydrolyzed collagen in combination with 50 mg vitamin C, 1 h before loading exercises, resulted in an increase in whole-body collagen synthesis and increased mechanics and collagen content of human-engineered ligaments [9]. These data suggest that supplementing hydrolyzed collagen and vitamin C can increase collagen synthesis. This is also hypothesized by Holwerda and Van Loon [10]. They state that mechanical loading as well as dietary intake of glycine and proline (main amino acids present in collagen) could stimulate mRNA transcription and hydroxylation of lysines and prolines, leading eventually to the development of a tropocollagen molecule. The final step, the crosslinking of the tropocollagen into a mature collagen molecule, is thought to be done by ascorbic acid, also known as vitamin C. Based upon this, we hypothesized that collagen and vitamin C in addition to the PTLE program (mechanical loading) might boost the synthesis of collagen, and therefore also the regeneration and recovery of the patella tendon. A recent randomized clinical trial showed that oral supplementation of specific collagen peptides may indeed accelerate the clinical benefits of a well-structured calf-strengthening and return-to-running program in Achilles tendinopathy participants [8]. A case study and explorative prospective phase IV cohort study investigating a similar intervention in athletes with PT also showed promising results [11, 12]. To our knowledge, the effectiveness of oral supplementation of specific collagen peptides in combination with vitamin C and PTLE in athletes with PT has not been studied in a RCT so far. Therefore, the research question of this study is: Is the use of oral supplementation of hydrolyzed collagen/vitamin C in addition to usual care (education, load management advice and PTLE) superior to usual care and placebo on the recovery of PT?

### Objectives {7}

The primary objective of this RCT is to evaluate whether the use of oral supplementation of hydrolyzed collagen/vitamin C in addition to usual care (education, load management advice and PTLE) is superior to usual care and placebo on Victorian Institute of Sports Assessment Patellar Tendinopathy (VISA-P) score (which rates pain, function, sports participation) after 24 weeks for athletes with PT. The secondary objective of this RCT is to evaluate whether the use of oral supplementation of hydrolyzed collagen/vitamin C in addition to usual care is superior to usual care and placebo with regard to other clinical outcome parameters, functional tests, and ultrasonographic tendon structure after 12 and 24 weeks for athletes with PT.

### Trial design {8}

**Fig. 1:**
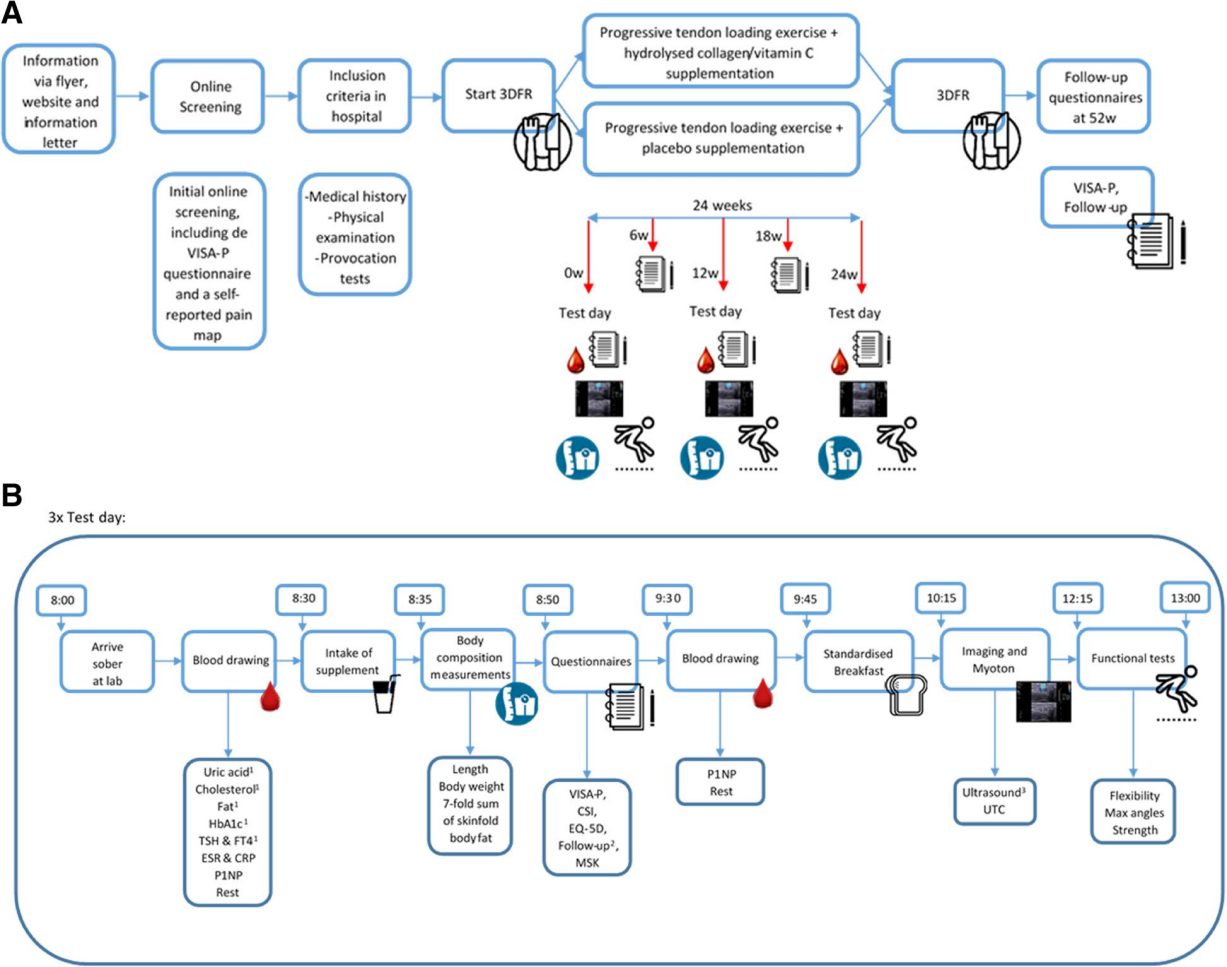
**A** Timeline of the study with measurements indicated per time point. Abbreviations: VISA-P, Victorian Institute of Sport Assessment—Patella; 3DFR, 3-Day Food Record. **B** Timeline of the test day with measurements indicated per time point. ^1^These blood parameters will only be measured on the first day. ^2^Follow-up questionnaire consists of subjective participant satisfaction with the PTLE program, the return to sports, the pain during activities of daily living, during sports and during the PTLE program, the use of co-interventions, training load, and the compliance of the supplement intake and the PTLE program. The follow-up questionnaire also contains the OSTRC (Oslo Sports Traumatic Research Centre questionnaire) and the SARS (Sports Activity Rating Scale questionnaire). ^3^The ultrasound (grayscale and power Doppler) will be performed at baseline and at 24 weeks. Abbreviations: ESR, (erythrocyte) sedimentation rate; CRP, C-reactive protein; CSI, Central Sensitisation Inventory; EQ-5D, EuroQol-5D-5L; FT4, free thyroxine; Hb1Ac, hemoglobin 1AC; MSK, Keele Start Back-Screening Tool; P1NP, procollagen type 1 N-terminal propeptide; TSH, thyroid-stimulating hormone; UTC, ultrasound tissue characterization; VISA-P, Victorian Institute of Sport Assessment—Patella

The JUMPFOOD study is a double-blinded, 2-armed randomized-controlled superiority trial (RCT). The duration of the intervention is 24 weeks after inclusion, with an additional follow-up questionnaire at 52 weeks (see Fig. 1A, B). Test days will take place at weeks 0, 12, and 24, with the first test day marking the start of the intervention period. Randomization will be performed as block randomization with a 1:1 allocation. The JUMPFOOD study will take place from February 2023 and is expected to finish at the end of 2025.

This study is registered in the National Institutes of Health (NIH) clinical trials registry (www.clinicaltrials.gov) prior to recruitment (NCT05407194). The study design, procedures, and informed consent procedure were approved by the Medical Ethics Committee (METC Oost-Nederland) of the Radboud UMC, the Netherlands (NL79100.091.22). All participants have to provide written informed consent.

## Methods: participants, interventions, and outcomes

### Study setting {9}

The study will be performed and the data will be collected and handled at the Sports Medicine department of the Gelderse Vallei Hospital in Ede, The Netherlands.

### Eligibility criteria {10}

In order to participate in this study, a participant must meet all of the following criteria: a clinical diagnosis of patellar tendinopathy based on a history of focal knee pain in patellar tendon or its patellar or tibial insertion in association with training and/or competition, palpation tenderness to the corresponding painful area (on the patellar tendon), and/or focal patellar tendon pain during patellar tendon loading with a pain provocation test (single leg decline squat and/or single leg jump squat, NB: imaging findings collected in this project will not be used as an eligibility criterion). Additional criteria are as follows: age 16–40 years old (the chosen age range will minimize chances of other conditions causing anterior knee pain such as osteoarthritis and osteochondrotic diseases like Sinding-Larsen-Johansson syndrome and Osgood-Schlatter disease), a current symptom duration of at least 12 weeks, sports participation at least once a week for at least 1 year before start of complaints, a VISA-P score < 80 out of 100 points, willingness to take (non-vegetarian) nutritional supplements, and willing and able to perform the PTLE program.

Athletes will be excluded if they have a known presence of inflammatory joint diseases (e.g., spondylarthropathy, gout, or rheumatoid arthritis) or familial hypercholesterolemia, if they underwent patellar tendon surgery, if they had a previous patellar tendon rupture of the index knee, if they suffer from acute knee injuries, including patellar tendon injuries with an acute onset, or if they show signs or symptoms of other coexisting knee pathology on physical examination (such as patellofemoral pain syndrome, joint effusion, and joint line tenderness), or if previously performed diagnostic imaging before the start of the study already showed other knee pathology (e.g., chondral lesion of the patella or trochlea on MRI or prepatellar bursitis on US). Athletes who use drugs with a putative effect on the patellar tendon in the preceding year (e.g., fluoroquinolones and statins); athletes who underwent local injection therapy with corticosteroids, other drugs, blood, platelet-rich plasma, or stem cells in the preceding 12 months; athletes who already participate in other concomitant treatment programs or research projects; athletes giving blood donation in a period of 2 months prior to each test day; athletes being pregnant or wish to become pregnant in the upcoming year; athletes who abuse hard drugs; athletes with an alcohol consumption > 21 units/week (men) or > 14 units/week (women); and athletes who already use hydrolyzed collagen supplementation will also be excluded.

### Who will take informed consent? {26a}

The executive researcher will take informed consent. Information about patellar tendinopathy and the purposes and procedures of the study will be presented on a specifically designed website. Athletes with knee pain who are informed about the study will be contacted with regard to possible questions they might have before signing informed consent. After both the participant and the executive researcher have signed the consent, the participant will be asked to fill out an online questionnaire for an in- and exclusion criteria check. After digital screening, those who meet the inclusion criteria will be invited for a clinical screening examination by an experienced sports physician.

### Additional consent provisions for collection and use of participant data and biological specimens {26b}

Additional blood samples will be obtained to be stored for use in future studies evaluating the pathobiology of PT. Information on the usage of blood samples is included in the current informed consent in order to specifically address the collection of these serum and plasma samples.

### Interventions

#### Explanation for the choice of comparators {6b}

All participants will receive education and advice with regard to load management and criteria-based PTLE. The latter consists of 4 stages within the limits of pain according to the multidisciplinary Dutch guidelines for anterior knee pain [13] and, thus, is part of the usual care. PTLE has recently been proven to be superior to eccentric training [6]. Participants will follow this PTLE program until full recovery. This can be sooner than the 24-week intervention. In that case, participants will keep on performing maintenance exercises from the PTLE program.

In addition to the PTLE exercise program, participants will be randomly assigned to receive either the nutritional supplement hydrolyzed collagen/vitamin C (intervention) or a placebo supplement for 24 weeks. Even if full recovery is reached within the 24 weeks of intervention, participants will complete the 24 weeks of supplement usage.

#### Intervention description {11a}

##### Hydrolyzed collagen + vitamin C versus placebo supplement

All participants will receive either a hydrolyzed collagen/vitamin C (intervention) supplement or a placebo supplement. The supplements will be taken per day for a duration of 24 weeks. Each day, the supplement will be taken 1 h before performing exercises from the PTLE program, or, when no exercises have to be performed, in the evening after dinner. The intervention supplement as well as the placebo are both produced by the company Rousselot BV (Ghent, Belgium). The intervention supplement contains 10 g of hydrolyzed collagen, in the form of collagen type I in combination with 40 mg vitamin C (48 kcal). The placebo supplement consists of 10 g maltodextrin (46 kcal). Both intervention and placebo powder are presented as sachets and the powder can be dissolved in cold water. The product is slightly sweetened to mask differences between intervention and placebo supplement.

##### PTLE (progressive tendon loading exercises)

All participants will receive criteria-based PTLE consisting of 4 stages within the limits of pain for 24 weeks. This intervention has recently been proven to be superior to eccentric training [6]. The program has been developed to be performed at the gym or in physical therapy practices. However, it has been modified so that participants can also perform the exercises at home, without equipment. The PTLE will be embedded in the usual care and is a method that is already implemented in many physical therapy practices. The exercise treatment will be explained to the participants by the executive researcher, together with physical therapists who received an explanation about the protocol before the study starts. In addition, the executive researcher will provide written information sheets and will provide a link to video instructions as part of the education. This includes an explanation of the condition, expected management, the positive influence of exercise therapy, and the positive effects of a gradual return to sports. Specific attention will be given to the relation between load and pain using the pain-monitoring model. Modification of all athletic activity (intensity, duration, frequency, and type of load) will be advised for activities that result in considerable patellar tendon pain, namely either significant reduction or even avoidance of the activity. We will stimulate the participants to perform (sports) activities within the limits of acceptable pain (VAS score ≤ 3 points on a scale 0–10, with 0 meaning no pain at all and 10 meaning unbearable pain).

The participants will need to follow an exercise program consisting of isometric (static), isotonic (dynamic), energy-storage (explosive), and sport-specific exercises consecutively, within the limits of acceptable pain [3]. Progression to each subsequent stage is defined using individualized progression criteria, based on the level of pain experienced during a pain provocation test that consists of one single-leg squat. If the VAS-score is 3 or less and exercises of the stage are performed for at least 1 week, progression to the next stage is advised.

Participants are instructed to gradually return to sport-specific training, performed every 2–3 days to allow for recovery from high tendon-loading exercises. In this stage, the isometric exercises of stage 1 will be continued on days that the sport-specific exercises are not performed. When all the exercises in stage 4 are performed within the limits of acceptable pain (VAS score ≤ 3 points), return to competition is recommended. Maintenance exercises are advised when return to play has been achieved. This includes isotonic exercises 3 times weekly and isometric exercises before training or competition.

As mentioned, the first instructions at the start of the intervention period will be given by the executive researcher and a physical therapist. After the elaborate instructions and explanations, participants will follow the exercise program, when possible under the guidance of a physical therapist, as part of their usual care program. Otherwise, the exercise program will be executed at a fitness center or at home. For all situations, instructional videos and an information brochure with explanations and pictures about the exercise program will be provided on a website. During the entire study period, a sports physical therapist with experience in tendon treatment will be available for participants when having questions.

The materials that are needed for home-based completion of these exercises (dynabands) will be offered to the participants after the instruction of the exercise program. The full PTLE program has been published elsewhere and is openly accessible [6].

#### Criteria for discontinuing or modifying allocated interventions {11b}

Participants can withdraw from the study at any time for any reason if they wish to do so without any consequences. The investigator can decide to withdraw a participant from the study for urgent medical reasons.

Since there are no further risks for participants in the study, there are no criteria for withdrawal of the participants based on any risk.

No adverse effects of hydrolyzed collagen or placebo are expected. Hydrolyzed collagen and maltodextrin are generally accepted as safe and have been used in this dosage and marketed in foods and dietary supplements globally for many years. In addition to the supplement, both groups are expected to experience improvement in symptoms as a result of the exercise therapy. In addition, the study has been designed to blend as much as possible with standard clinical care.

#### Strategies to improve adherence to interventions {11c}

Before the start of the intervention, participants will receive an elaborative explanation about the supplement and the exercise program. The explanation is also provided on the website, including short video clips with the exercises and a pdf containing all information about the exercise program.

Two factors will be monitored to keep track of study adherence: (1) compliance to the supplement intake (number of empty and unused supplement sachets brought by the participant and via the weekly questionnaire) and (2) compliance to the exercise program (total percentage of prescribed exercises performed (amount of load and number of series and repetitions will be monitored)). This will be asked on a weekly basis using a short online questionnaire. These questionnaires will be checked on a weekly basis. When the questionnaire is not filled out on the designated day, a reminder will be sent. With no response, a day later, participants will be called to remind them about the questionnaire.

#### Relevant concomitant care permitted or prohibited during the trial {11d}

Pain relieving medication should preferably not be used, but when participants experience pain with a VAS score of 7 or higher, with a duration of at least 24 h, the use of pain medication will be allowed. Preferably, participants should only use paracetamol (acetaminophen) as pain medication. Long-term use of NSAIDs is not allowed. All pain medication should be reported with the type of pain medication, dosage, date of intake, and reason of intake (e.g., knee pain or other causes of pain). No pain medication is allowed the day before and on the day of the tests. Participants have to report all use of co-interventions (patellar strap, inlays, pain medication, or other) in their weekly diary, as the use of pain-relieving therapies is a variable in this study.

#### Provisions for post-trial care {30}

If participants still experience symptoms after completion of the study, we will advise them to contact the general practitioner for a referral to a sports medicine physician.

### Outcomes {12}

#### Primary outcome measure

##### VISA-P questionnaire

The primary outcome measure of the JUMPFOOD study is the change of the validated and disease-specific VISA-P score over 24 weeks, which will be compared between the supplement and placebo groups. This is a short questionnaire specifically designed for athletes with patellar tendinopathy measuring the severity of the injury by assessing pain, function, and ability to participate in sports activities [14]. A VISA-P score of 100 indicates no pain, maximum function, and maximum ability to play sports. The score decreases with increasing severity of symptoms of PT. The VISA-P questionnaire has been translated and validated in Dutch language [14]. The responsiveness of the VISA-P scale has recently been established in athletes with patellar tendinopathy. The minimum clinically important difference (MCID) of the VISA-P score was 13 points in that study [15].

#### Secondary outcome measures

##### Body composition measurements

Body weight (kg), height (cm), and body fat percentage (%) using the sevenfold sum of skinfold will be measured.

##### Blood sampling

At all three test days (*t* = 0, *t* = 12, and *t* = 24 weeks), one blood sample will be drawn at baseline and one will be drawn 1 h after intake of the intervention or placebo supplement. At all test days, a sober blood sample will be drawn for the analysis of BSE and CRP which are general parameters for the determination of inflammation [16, 17]. At only the first test day, uric acid [18–23], cholesterol and lipid concentration [17, 24–28], Hb1Ac [26, 29, 30], thyroid-stimulating hormone (TSH), and free thyroxine (FT4) will be measured, since elevated levels are linked with a higher prevalence of tendon complaints, and thyroid hormones play a role in the etiology of tendinopathy [24, 25, 31].

At all three test days, at baseline and 1 h after intake of the supplement, a blood sample will be drawn to determine the specific N-terminal peptide of pro-collagen I (PINP) concentration change due to supplement intake and change over time to determine collagen build up in the body.

#### Functional tests

##### Functional tests

Functional tests will be performed to determine the pain rating on a visual analogue scale (VAS) and to determine flexibility and strength. First, the participant has to perform a single leg decline squat where the participant performs a single leg squat to 60° of knee flexion on a 25° decline board. This test was designed to preferentially load the patellar tendon [32–35]. We record the VAS pain score after the first squat and after ten single-leg squats on the decline board. Isometric strength of the quadriceps, hamstrings, and abductors will be measured using the MicroFET (ProCare, Groningen, the Netherlands). Muscle length of the quadriceps and the hamstrings will be measured using a plurimeter. Also, a weight-bearing dorsiflexion lunge test will be performed to measure the angle of dorsiflexion using a plurimeter. Using the OptoGait (ProCare, Groningen, the Netherlands), we will measure ground reaction time during a drop jump test, we will measure the ratio between the tendon and muscle strength during a squat jump vs countermovement jump, and we will measure the difference and ratio between the left and right knee during hop tests (single, triple, medial side triple, 90° medial rotation and side hop test). Finally, a drift test will be performed to measure ground area deviation. All functional tests will first be performed on the contralateral side, followed by the affected side. If both sides are affected, the most affected side will be registered as affected side.

#### Imaging

##### Imaging

*Ultrasound* (Hitachi Aloka Arietta 850 machine, with 18–5 Hz linear echo probe, in B mode 25 fps, in Doppler 19 fps, Tokyo, Japan) of the patellar tendon will be performed by an experienced musculoskeletal radiologist, or under the supervision of an experienced musculoskeletal radiologist. The participant will be examined in the supine position with the knees flexed for determining the structure and extended for determining vascularization. The ultrasound scan includes an assessment of patellar morphology and echogenicity and Power Doppler assessment (PDU) of the vascularity of the tendon. All mentioned aspects can be measured by performing one ultrasound per knee.

*Ultrasound tissue characterization (UTC)* of the patellar tendon will be used to determine 3D tendon integrity. Structure of the patellar tendon will be quantified using UTC. A standardized protocol will be used to take the UTC scans [36]. Participants will sit down on a treatment bench with the bench as much as possible in the popliteal fossa with approximately 90° knee flexion. An ultrasound probe (SmartProbe 12L5-V, Terason 2000 + ; Teratech) will be affixed to a tracking device (UTC Tracker, UTC Imaging) to standardize transducer tilt angle. The tracker device will move the probe automatically over the length of the tendon, capturing transverse images every 0.2 mm. The ultrasound probe in the tracking device will be placed perpendicular to the long axis of the tendon, moving from proximal to distal. Both patellar tendons will be scanned for each participant. All scans will be taken by a single examiner who is experienced with UTC. The consecutive transversal images will be used to create 3D reconstructions. The consistency of intensity and distribution of gray images will be calculated over a distance of 4.8 mm using UTC algorithms. Four echo types can be discriminated based on consistency, with echo type I representing the most stable pattern and echo type IV the least stable. Tendon structure will be quantified by calculating the percentages of these four echo types in multiple regions of interest (ROI) placed around the border of the tendon in the transverse view. ROIs will be selected at intervals no greater than 5 mm from the apex of the patella to 20 mm distally. Contours will be drawn every 5 mm and window size 17 will be used for imaging analysis. All tendons contours will be marked by trained personnel, due to practical implications. The inter- and intra-rater reliability of the processing of the scans is found to be excellent [37, 38].

##### Tendon stiffness

Patellar tendon stiffness will be assessed using myotonometric measurements with the MyotonPRO device (Myoton Ltd, Tallinn, Estonia). The measuring method of this device is based on the free oscillation technique. The following steps occur during a single measurement: (1) the device is placed perpendicular to the skin and a constant pre-pressure is applied for compressing the subcutaneous tissue; (2) a brief mechanical impulse is applied causing damped oscillation of the tissue; and (3) the co-oscillation generated in the tissue is recorded by an accelerometer. The myotonometer has good to excellent test–retest reliability and has been established in previous literature [39].

The anatomical landmark for the patellar tendon stiffness assessment will be the most painful spot in the patellar tendon but also 1 cm below the inferior pole of the patella and the midpoint of the patellar tendon. The participants will lie down on a treatment bench with their knees flexed to 90°, measured with a goniometer. In addition to stiffness measurements on the tendon, the stiffness of the rectus femoris muscle will be measured as well, at 1/3 and 2/3 of the muscle, with extended hip and knee. The myotonometric evaluation will be repeated 3 times per location. Only measures that have a coefficient of variation lower than 3% will be considered; otherwise, the measurement will be repeated.

For all imaging techniques and tendon stiffness measurements, the contralateral patellar tendon will also be examined for comparison. Here as well, if both sides are affected, the most affected side will be registered as the affected side.

##### Nutrition

A 3-Day Food Record (3DFR) will be recorded at the start and at the end of the intervention period by the use of the validated TRAQQ app of Wageningen University [40, 41]. Using this app, participants report what they have eaten for 3 days, after which the app automatically calculates the macro- and micronutrient intake. These 3 days will take place on two workdays and one weekend day. This will be measured to investigate their current nutritional status and potential causes or relationships between nutrition and the current tendon problems. Nutrients that will be measured are among others: protein and amino acid intake, total energy intake, alcohol consumption, and vitamin C intake. These nutrients were found to affect tendon health in a recent systematic review [42]. Amino acid intake is a more specific item that can be included by the use of the amino acid table that is part of the TRAQQ app and the Wageningen University. Here, the amino acid content of products is added to the Dutch food composition database. During the entire intervention period, participants will be asked not to radically change their diet. For example, changing to a vegan diet is not allowed during the study if this was not the participant’s habitual diet before the start of this study.

##### Questionnaires

Multiple questionnaires will be given at baseline, at 12 weeks, and at 24 weeks. These questionnaires involve general health and well-being (ED-5Q), central sensitization of pain (CSI), the influence of the participants’ injury on sports participation (OSTRC), categorizing subgroups within patients with PT (MSK), and a sports activity rating scale (SARS). In addition, a follow-up questionnaire will be given investigating subjective participant satisfaction with the PTLE program, the return to sports, the pain during activities of daily living, during sports and during the PTLE program, and investigating the use of co-interventions, training load, and the compliance to the supplement intake and PTLE program.

#### Other outcomes

##### Weekly logbooks

Data on compliance to the training program, mean pain score during the exercises, and sport-specific training load will be registered on a weekly basis. The questionnaire will be automatically sent weekly as a reminder. In this online questionnaire, we ask the number of training days that week and the number of matches that week. We ask which percentage of the prescribed repetitions the participant had accomplished and the precise amount of load and number of series and repetitions of the exercise program executed. The training load that will be obtained from this questionnaire is defined as the total duration of training and matches played per week (minutes) multiplied by the experienced intensity of training sessions and games using the modified Borg CR-10 Rate of Perceived Exertion (RPE) scale [43]. In addition, we ask the level of pain when performing the exercises, and we ask for reasons why the participant has not performed the prescribed exercises. Answering options are (1) lack of time, (2) pain, (3) preferring sports activities, (4) rest day, and (5) lack of motivation. We ask whether the participant has returned to their sports level or not; answering options are (1) not active in sports, (2) no return to sports, (3) returning to sport but not in desired sport, (4) returning to desired sport but not at the preinjury level, and (5) returning to preinjury level in the desired sport. We ask how satisfied the participant is with the exercise program; answering options are (1) excellent, (2) good, (3) moderate, and (4) poor.

At the end of the study, participants will be asked whether they knew which of the two supplements they received during the intervention period. The results of the weekly questionnaire are stored using a secure data collection site where data can be accessed, submitted, and modified.

### Participant timeline {13}

Participants will be recruited from April 2023 until May 2025. Upon registration and signing informed consent, the executive researcher will screen participants for potential eligibility. To prevent participants from making an unnecessary visit to the hospital, the PI will ask among others the age, absence of known inflammatory diseases or hypercholesterolemia, previous knee conditions and treatments, use of medication, and participation in other treatment programs. To increase the likelihood of the diagnosis of patellar tendinopathy, we will use a self-administered pain map, where one of the six images shows the situation of PT. The potential participant will finally complete an online VISA-P questionnaire, which should be below 80 points to be eligible for the study. If these criteria are met, the participant is invited for inclusion measurements at the hospital Gelderse Vallei.

When the participant arrives at the hospital, further screening for inclusion criteria follows. A clinical examination will be performed by an experienced sports physician. The diagnosis will be based on clinical examination: focal pain at the inferior patellar pole or patellar tendon on palpation and a single-leg-decline squat. If the potential participant meets all the inclusion criteria, he/she is allocated to either intervention or placebo and invited for another appointment at the hospital for the baseline measurements. In addition, the 3DFR will be scheduled. This will be explained, which the participant fills in at home, using the app “TRAQQ” of Wageningen University.

At baseline, a test day will take place at hospital Gelderse Vallei, containing the outcome measures stated above (blood measurements, body composition measurements, questionnaires, imaging, stiffness measurements, and functional tests; see Fig. 1B and Table 1). At follow-up (12 and 24 weeks), the clinical, radiological, and blood parameters will be repeated. The measurements will be performed in an identical way as during the baseline measurements, with the exception of the ultrasound, which is only performed at baseline and at 24 weeks (see Fig. 1B). In addition, the 3DFR will be obtained at baseline and after 24 weeks, with no nutritional assessment at 12 weeks.

**Table 1:** Schematic diagram of the JUMPFOOD study

		V1		V2–V4			
	At home by mail or telephone	At the hospital Sports Valley	Start of study	At the hospital Sports Valley Week 0, week 12, and week 24	Online Week 6, week 18, and week 52	During the entire 24 weeks, daily	During the entire 24 weeks, weekly
Handover study information and informed consent	X						
Telephone call: discuss questions about participation	X						
Signing informed consent	X						
Online screening	X						
VISA-P	X						
Pain map	X						
Inclusion questions	X						
Hospital screening		X					
Physical examination		X					
Ultrasound		X					
Functional tests		X					
Explanation PTLE program		X					
Randomization			X				
Study measurements				X			
Body composition^b^				X			
Questionnaires^c^				X	X^a^		
Blood sampling				X			
Functional tests^d^				X			
Imaging^e^				X			
Myoton				X			
Compliance (sachets check)				X			
Supplement use						X	
PTLE program (3 times/week)^f^							X
Compliance							X

^a^Only the VISA-P is asked digitally in weeks 6, 18, and 52

^b^Consisting of length and body weight measurements and sevenfold sum of skinfolds measurements

^c^Consisting of the VISA-P, EQ-5D, MSK, CSI, SARS, OSTRC, and follow-up questions investigating satisfaction, return to sports, pain during activities of daily living, during sports and during the PTLE program, the use of co-interventions, compliance to exercise program and supplement intake, and investigating training load

^d^Consisting of the single leg decline squat and measurements of the isometric strength of the quadriceps, hamstrings, and abductors; muscle length; drop jump test; weight-bearing dorsiflexion lunge test; hoptests; and the squat vs countermovement jump

^e^Consisting of an ultrasound (grayscale and power Doppler) and UTC, with the ultrasound executed at 0 and 24 weeks and not at 12 weeks

^f^Part of usual care

Tendinopathy-related symptoms will be recorded at baseline and 6, 12, 18, 24, and 52 weeks’ follow-up (Fig. 1A) using the VISA-P score. At 6, 18, and 52 weeks, this outcome measurement will be determined using a web-based form. The follow-up questionnaire that is given at baseline and 12 and 24 weeks is also given at 52 weeks of follow-up.

### Sample size {14}

The mean difference of change in VISA-P score over time between the intervention and placebo group is estimated to be 13 points higher in the intervention group compared to the placebo group. The standard deviation of the difference in VISA-P score is estimated to be 19 [15]. With a power estimated to be 80% and a significance level of 0.05, the sample size calculation (performed in G*Power 3.1.9.7) led to a total sample size of 70 participants. As we will compensate for an expected loss to follow-up of 9%, 76 participants will be included in total, i.e., 38 participants per treatment arm.

### Recruitment {15}

Dutch male and female athletes (age 16–40) suffering from anterior knee pain will be informed about the JUMPFOOD study via physical therapists, sports associations, clubs, and sport physicians. Coaches and clubs within the region of the study location are also informed about the study. Additionally, local, regional, and national publicity campaigns are used to draw attention to the study. For example, the Dutch volleyball association (NeVoBo), basketball association (NBB), handball association (NHV), korfball association (KNKV), and athletics association (Atletiekunie) will be informed about this study, and athletes will be informed through their websites and newsletters.

## Assignment of interventions: allocation

### Sequence generation {16a}

Participants will be randomly assigned to either the intervention or the control group using a block randomization with blocks of 8 participants. Blocks will be based on the date of entry. The randomization list will be prepared by an independent person, not associated with the study. This person is blinded to information about the study participants. This independent person works at a different department of the Wageningen University & Research (WUR) and will only be involved in the randomization and blinding of this study. This person will not be involved in further steps or analyses of the JUMPFOOD study. Randomization of participant numbers to supplement numbers will be performed using a random number generator.

### Concealment mechanism {16b}

The allocation sequence containing the supplement numbers linked to the treatment is in a sealed “emergency code envelope.” During the entire study, the executive researcher only has a list of participant numbers and supplement numbers. The supplement number cannot be traced back to either the supplement or the placebo group.

### Implementation {16c}

The allocation list containing supplement numbers linked to participant numbers is in possession of the executive researcher, who will allocate participants to participant numbers during enrolment. The assignment to interventions is blinded and only known by the independent person who performed the randomization beforehand. The executive researcher therefore also does not know which participants are in the same group (placebo or intervention).

## Assignment of interventions: blinding

### Who will be blinded {17a}

All participants, care providers, outcome assessors, and data analysts will be kept blind to the assignment of the type of intervention. Only the independent randomization person, who is not involved in further steps or analyses of the study, knows the treatment allocation. Blinding of the hydrolyzed collagen/vitamin C supplement is possible since the placebo supplement will look, smell, and taste approximately the same. After data analysis, the codes will be broken.

### Procedure for unblinding if needed {17b}

The executive researcher will receive the code of the independent randomization person identifying which treatment the participant receives in case of an emergency in which a participant will have to be unblinded.

## Data collection and management

### Plans for assessment and collection of outcomes {18a}

Each researcher involved in this study will be trained in the study requirements; standardized measurement of height, weight, and fat percentage; how to handle the myotonometer, caliper, UTC, OptoGait, Castor, and questionnaires; how to prepare the supplements; and how to explain everything and guide the participants during study participation.

During study execution, all data that will be obtained will be filled out in Castor with additional checks within a day. On all test days, the painful knee will be marked in the morning, so every researcher knows which leg the affected leg is. Tests are first performed on the unaffected knee and thereafter on the affected knee.

For further information, see the “Outcomes {12} section.”

### Plans to promote participant retention and complete follow-up {18b}

Participants will receive their own data every test day to promote participant retention and complete follow-up. In addition, newsletters will be shared among participants showing their personal details with further explanations, and fact sheets will be shared showing the current study status and results.

### Data management {19}

Data collection will be performed using the electronic data capture system Castor. All assessors performing the measurements will be intensively trained beforehand, if not already trained by education (radiologist performing ultrasound and qualified nurses performing blood sampling). Functional tests and stiffness measurements will be performed twice or three times to promote data quality. In addition, further data quality is promoted by the use of reliable and valid measurements and questionnaires as mentioned above.

### Confidentiality {27}

Personal data of the participants that will be collected during the present study will be handled confidentially and coded, in compliance with the General Data Protection Regulation (GDPR) (Dutch: Algemene Verordening Gegevensbescherming, AVG). Included participants will be identified by an anonymous identification code which is not based on the participants’ initials, name, or date of birth. Participants will be given a unique code (10.001, 10.002, 10.003, etc.). This code is linked with the name, date of birth, and contact information of the participant in a protected file, and only the members of the research team can access this file. This study number will be used for all study documentation and all study reports or publications. Where it is necessary to be able to trace data to an individual participant, a participant identification code list can be used to link the data to the participant. Data will be stored on a secure server at ZGV for 15 years in order to be used for further analyses in line of this research. Blood samples obtained during the study will be stored for 5 years using the unique codes. The handling of personal data will comply with the GDPR. When participants give consent for participation, they also give consent for storage of their data. Study results will be published in a peer-reviewed journal.

The METC Oost-NL will be consulted when new research is carried out with the residual material taken during this current study, during the next 5 years, with the possibility of new findings about the health status of the donors.

### Plans for collection, laboratory evaluation, and storage of biological specimens for genetic or molecular analysis in this trial/future use {33}

For future analyses, we will take extra blood at baseline and 1 h after intake of the supplement, at all three test days. This blood sample will be saved for future analyses, because the results of previous measurements may give rise to follow-up measurements. For example, results for pro-collagen-1 concentrations might be a reason to subsequently test for specific amino acids or growth factors. New insights may lead to specific cytokine analysis. In addition, in an in vitro study we want to add the collected blood samples to tendon cells.

## Statistical methods

### Statistical methods for primary and secondary outcomes {20a}

Statistical analyses will be performed with SPSS software (Version 27, IBM, Armonk, NY, USA). Collected data will be checked for completeness and normality of distribution (using the Shapiro–Wilk test). For the normally distributed variables, the parametric test will be used. For the variables that are not normally distributed, the non-parametric test will be used. Normally distributed continuous variables will be presented quantitatively as mean (± SD), or graphically with mean (± SD). Data that is not normally distributed will be presented as median and interquartile range. Categorical data will be presented as percentages. A *p*-value of ≤ 0.05 will be considered statistically significant.

The primary study parameter will be the difference between intervention and placebo in the change in VISA-P score (0–100) over time. The primary outcome will first be analyzed with a linear mixed model. When the residuals of the model are not normally distributed, we will use a generalized linear mixed model instead. As independent variables, the treatment (supplement or placebo), time (time points of the measured variable) and time × treatment will be fixed factors, and the participant will be used as a random factor. Adjustments will be made for those baseline variables that are associated with the primary outcome (*p* < 0.10), using a multivariable analysis and a stepwise backward elimination strategy. Examples of potentially associated variables are baseline vitamin C and collagen amino acid intake, baseline P1NP concentrations, compliance to PTLE program, compliance to supplement use, and use of co-medication. Secondary study parameters will be reported quantitatively as well, in the most commonly used way. For the secondary study parameters, we will choose between the generalized linear mixed model and the linear mixed model in the same manner as for the primary outcome. Here, as independent variables, the treatment (supplement or placebo), time (time points of the measured variable), and time × treatment will be fixed factors, and the participant will be used as a random factor. The dependent variable is the secondary study parameter in question (with as important dependent variables/outcome measures: pain during a single leg decline squat, ultrasound imaging abnormalities, UTC findings, and tendon and muscle stiffness). The same stepwise backward elimination strategy will be used as mentioned above. For parameters that are categorical (dichotomous, nominal, or ordinal) a generalized linear mixed model will be used that is applicable for that specific secondary variable (for example a logistic mixed model for dichotomous parameters, and an ordinal mixed model for ordinal parameters). For these parameters, a similar strategy will be applied as described above.

### Interim analyses {21b}

Not applicable, as this trial has no DMC, the duration of recruitment is manageable and there are no potentially serious outcomes.

### Methods for additional analyses (e.g., subgroup analyses) {20b}

At the moment, additional analyses are not foreseen; however, in the future, we might analyze the difference between different sports, the difference between proximal or distal injury on the tendon, and the difference between affected and unaffected tendons in tendon structure over time.

### Methods in analysis to handle protocol non-adherence and any statistical methods to handle missing data {20c}

Consistent with the CONSORT statement, an intention-to-treat analysis will be performed. In order to ensure the integrity of the randomization process and to offer an authentic assessment of the treatment outcomes, we will implement the intention-to-treat principle, analyzing the entire study population irrespective of their adherence to the treatment. We will carry out sensitivity analyses to assess the influence of missing values in the outcome parameter on the results. Then, we will decide on a strategy on how to handle the missing data if necessary. If data is missing “completely at random” (MCAR), we will perform multiple imputation to model the missing data within the intention-to-treat analysis, thus preserving the variability inherent in the outcome variables [44–46].

### Plans to give access to the full protocol, participant-level data, and statistical code {31c}

We plan to write at least one scientific paper about the results from the JUMPFOOD study. This will be published (if accepted) in a peer-reviewed journal. In this manner, we plan to share our findings with the world. In combination with the registration in the Clinical Trials register and this design paper, the full protocol is already publicly accessible. The anonymous datasets analyzed during the current study and the statistical codes will be available from the corresponding author upon reasonable request after finalizing the papers necessary for completing the doctoral degree of the corresponding author.

## Oversight and monitoring

### Composition of the coordinating center and trial steering committee {5d}

Principal Investigator:

Design and conduct of JUMPFOOD.

Preparation of protocol and revisions.

Preparation of investigators brochure (IB) and CRFs [Case Report Forms].

Organizing steering committee meetings.

Managing CTO [Clinical Trials Office].

Publication of study reports.

Steering committee (SC):

Design and agreement of final protocol.

Budget administration.

Executive researcher:

Design and conduct of JUMPFOOD.

Preparation of protocol and revisions.

Preparation of investigators brochure (IB) and CRFs [Case Report Forms].

Organizing and steering committee meetings (weekly).

Study planning.

Organization of test days.

Provide annual risk report to ethics committee (METC-OOST).

SUSAR [Serious unexpected suspected adverse events] reporting to METC-OOST.

Responsible for trial master file.

Organization of central serum sample collection.

Data manager.

Follow-up of participants.

Publication of study reports.

Randomization person:

Randomization.

Independent expert:

Answering questions related to study.

### Composition of the data monitoring committee, its role and reporting structure {21a}

Not applicable. A DMC is not needed in this study as risks are very low for the participants and it is a single-center study.

### Adverse event reporting and harms {22}

All adverse events reported spontaneously by the participant or observed by the investigator or his staff will be recorded. Although no serious adverse events are foreseen, these will be reported to the ethical committee within 7 days of first knowledge. All adverse events will be followed until they have abated, or until a stable situation has been reached. Depending on the event, follow-up may require additional tests or medical procedures as indicated, and/or referral to the general physician or a medical specialist. Serious adverse events need to be reported to the medical ethical committee till end of study within the Netherlands.

### Frequency and plans for auditing trial conduct {23}

Auditing trials are not conducted as this is a single-center study with low risk for the participants.

### Plans for communicating important protocol amendments to relevant parties (e.g., trial participants, ethical committees) {25}

When an amendment is wanted in the study protocol, this will be reported to the medical ethical committee (METC-OOST). Only after agreement from the METC will this amendment be definitive.

### Dissemination plans {31a}

We plan to publish at least one scientific paper about the results of the JUMPFOOD study. This will be submitted to a peer-reviewed Q1 sports medicine or sports nutrition journal. In this manner, we plan to share our findings with the world. The study results will be released to the participating physicians, referring physicians, participants, and the general medical community. Results will also be presented at (inter)national conferences and we strive to implement our findings in future treatment guidelines.

## Discussion

Despite the high prevalence of PT among athletes and its potential impact on sports participation, management of (patellar) tendinopathy remains challenging. Hydrolyzed collagen and vitamin C supplementation appears to have a promising effect on the recovery of (chronic) patellar tendinopathy in combination with exercise therapy. Positive effects of hydrolyzed collagen supplementation have been found on the recovery of Achilles tendinopathy and several studies suggest an increased collagen synthesis with hydrolyzed collagen supplementation [8, 9]. In addition, vitamin C might play a role in the final step, the crosslinking of the collagen molecule, showing its importance in tendon injury recovery [10]. However, to our knowledge, no RCT has been performed yet investigating the effect of hydrolyzed collagen and vitamin C supplementation on the recovery of patellar tendinopathy. While there are not many studies on nutrition and tendon health, this is the first RCT to study the effectiveness of hydrolyzed collagen/vitamin C supplementation in combination with the PTLE program on the recovery of patellar tendinopathy in athletes. This is also the first study with the PTLE program that takes into account the training and competition load of the athletes during the entire study period using a web-based log/app. The JUMPFOOD study is one of the largest RCTs on PT. Including two additional intervention arms to disentangle the separate effects of collagen and vitamin C would make the study design more robust, but this would require an unrealistically high sample size. In addition, researchers were forced to choose block randomization to have both placebo and intervention evenly distributed throughout the year. For example, in July and August, most athletes exercise less or differently compared to when all competitions start in September, influencing their experienced PT pain.

Another strength of the JUMPFOOD study is that it evaluates a variety of outcome measures of the patellar tendon, including imaging, functionality, stiffness, and pain, resulting in a better understanding of the effect of PTLE and collagen on injury and its recovery. In addition, the study is incorporated into usual care/Dutch guidelines which might facilitate implementation of the results. If supplementation of collagen and vitamin C appears to be effective, this treatment approach can be implemented in daily sports medicine practice to improve the treatment outcome of patients with PT. Overall, this study helps to better understand the role of collagen and vitamin C in addition to PTLE in the treatment of PT and leads us a step further in achieving better treatment results for this common and bothersome sports injury.

## Conclusion

The JUMPFOOD study is the first large RCT to evaluate the effectiveness of hydrolyzed collagen/vitamin C supplements in combination with a PTLE program on the recovery of the jumpers’ knee or PT.

### Trial status

Protocol version: 1

Date: 28–08-2023

Start date recruitment: 01–04-2023

End date recruitment: 01–05-2025

### Supplementary Information

**Additional file 1. **Informed consent form.

## Data Availability

Persons who have access to the final dataset are the research team members, the principal investigator, the project leader, and the executive researcher, as well as Rousselot. Blood samples for future analyses will be stored and only the research team members can access them.

## Abbreviations

3DFR: 3-Day Food Record
ANOVA: Analysis of variance
BMI: Body mass index
CONSORT: Consolidated Standards of Reporting Trials
CSI: Central Sensitization Inventory
EQ-5D: EuroQuol-5D-5L
FT4: Free thyroxine
GDPR: General Data Protection Regulation
MCID: Minimal Clinically Important Difference
METC: Medisch Ethische Toetsingscommissie (Medical Ethical Committee)
MSK: Keele Start Back-Screening Tool
NBB: Dutch Basketball Association
NeVoBo: Dutch Volleyball Association
NHV: Dutch Handball Association
KNK: Dutch Korfball Association
OSTRC: Oslo Sports Trauma Research Centre questionnaire
PDU: Power Doppler Ultrasonography
PINP: Pro-collagen I
PT: Patellar tendinopathy
PTLE: Progressive tendon loading exercises
RCT: Randomized controlled trial
SARS: Sports Activity Rating Scale
SD: Standard deviation
TSH: Thyroid-stimulating hormone
UTC: Ultrasound tissue characterization
VAS: Visual Analogue Scale
VISA-P: Victorian Institute of Sports Assessment—Patella
WUR: Wageningen University & Research
ZGV: Gelderse Vallei Hospital
