# Supplementary material for: The JUMPFOOD study: additional effect of hydrolyzed collagen and vitamin C to exercise treatment for patellar tendinopathy (jumper’s knee) in athletes—study protocol for a double-blind randomized controlled trial
Source: Trials. 2023 Nov 28;24:768. doi: 10.1186/s13063-023-07783-2 (PMC10685530; doi:10.1186/s13063-023-07783-2)
Supplement: Supplementary file 1 — Additional file 1. Informed consent form. [file 13063_2023_7783_MOESM1_ESM.docx]

**Informed consent form**

Belonging to JUMPFOOD study (The effect of gelatin and vitamin C in the treatment of 'jumping knee')

- I have read the information letter. I was also able to ask questions. My questions were answered well enough. I had enough time to decide whether to participate.
- I know that participating is voluntary. I also know that I can decide at any time not to participate in the study after all. Or to stop. I do not have to say why I want to stop.
- I give the researchers permission to inform my general practitioner and if applicable, physical therapist, about my participation in this study.
- I give the researchers permission to give my GP or specialist information about unexpected findings from the study that are important to my health.
- I give the researchers permission to collect and use my data and body material. The researchers do this only to answer the research question of this study.
- I know that for the purpose of monitoring the study, some people related to the study may see my data. Those people are listed in the information letter. I give these people permission to see my data for this audit.
- Would you please check yes or no in the table below?

| I give permission to keep my data for 15 years to use it for other research, as stated in the information letter. | Yes ☐ | No☐ |
| --- | --- | --- |
| I give permission to keep my (remaining) body material to use it for other research, as stated in the information letter. The body material will be kept for 5 years for this purpose. | Yes ☐ | No ☐ |
| I give permission to ask me if necessary after this study if I would like to participate in a follow-up study. | Yes ☐ | No ☐ |
| I give the researchers permission to let me know after the study which treatment I received/which group I was in. | Yes ☐ | No ☐ |

- I want to participate in this study

My name is (participant): ………………………………..

Signature: ……………………… Date : __ / __ / __

-----------------------------------------------------------------------------------------------------------------

I declare that I have fully informed this participant about the aforementioned study.

Will any information become known during the research that may affect the participant's consent? If so, I will let this participant know in time.

Name researcher (or their representative):……………………………….

Signature:……………………… Date: __ / __ / __

-----------------------------------------------------------------------------------------------------------------

Additional information was provided by:

Name:………………………………..

Function:………………………………

Signature:……………………… Date: __ / __ / __

-----------------------------------------------------------------------------------------------------------------

*A complete information letter will be given to the participant along with a signed version of the consent form.*
